# Sonographic image of cervix epithelioid trophoblastic tumor coexisting with mucinous adenocarcinoma in a postmenopausal woman: A case report

**DOI:** 10.1097/MD.0000000000007731

**Published:** 2017-09-22

**Authors:** Yi Zhu, Guo-Nan Zhang, Rui-Bo Zhang, Yu Shi, Deng-Feng Wang, Rong He

**Affiliations:** a Department of Ultrasound; b Department of Gynecologic Oncology, Sichuan Cancer Hospital & Institute, Sichuan Cancer Center, School of Medicine, University of Electronic Science and Technology of China, Chengdu, Sichuan, China.

**Keywords:** cervical cancer, epithelioid trophoblastic tumor, gestational trophoblastic disease, mucinous adenocarcinoma, ultrasound

## Abstract

**Rationale:**

Epithelioid trophoblastic tumor (ETT) is a distinctive but rare gestational trophoblastic neoplasia (GTN) composed of chorionic-type intermediate trophoblast cells. Approximately 50% ETT arose from the uterine cervix or lower uterine segment following a previous pregnancy with vaginal bleeding. With its unusual ability to simulate an invasive epithelioid neoplasm, ETT frequently poses a diagnostic challenge, especially involving the uterine cervix.

**Patient concerns:**

We herein report the case of a 60-year-old female with persistent vaginal bleeding and middle-level elevation of serum human chorionic gonadotropin (hCG). Ultrasound revealed a 3.0 × 2.7 cm well-circumscribed, strongly echogenic lesion in the cervix, with a peripheral pattern of Doppler signals. The enhanced pattern by contrast-enhanced ultrasound displayed strong peripheral enhancement accompanied with globular appearance, then centripetal filling completely, and fading away rapidly.

**Diagnoses:**

The final pathological diagnosis was ETT accompanying mucinous adenocarcinoma.

**Interventions:**

Due to the pre-operative evaluation of a presumed IB2 cervix mucinous adenocarcinoma, the patient was treated with 2 courses of neoadjuvant chemotherapy followed by radical hysterectomy.

**Outcomes:**

The patient is currently disease-free for the past 1 year.

**Lessons:**

This case report demonstrates that sonographic image of tumor shapes and blood flow could be helpful in differentiating ETT from another GTN and enable more accurate diagnosis before treatment.

## 1 Introduction

Epithelioid trophoblastic tumor (ETT) is an entity and originates from chorionic-type intermediate trophoblastic cells, which has only recently been identified and distinguished from other types of gestational trophoblastic neoplasm (GTN). ETT is liable to be chemoresistant, so that the primary choice of treatment is surgery intervention. However, because of its low incidence and limited knowledge in clinical practice, ETT has a great possibility of being confused with several trophoblastic and nontrophoblastic lesions, notably placental-site trophoblastic tumors (PSTTs) and invasive squamous carcinoma of the cervix. Thus, this may lead to progressive tumor development, metastasis, and poor prognosis. Pelvic ultrasound as the initial imaging investigation for gynecologic oncology, unfortunately, there were few reports of ETT focusing on sonographic image. The following case pays special attention to the sonographic features of cervix ETT accompanying mucinous adenocarcinoma in a postmenopausal woman. We also compared these sonographic findings with histopathological features to deduce their relativity, and identified distinctions from other GTNs and cervical cancer.

## 2 Methods

We collected this patient's medical records and reviewed the related literatures. Internal Ethical Committee at Sichuan Cancer Hospital approved this study.

## 3 Case presentation

**Figure 1:**
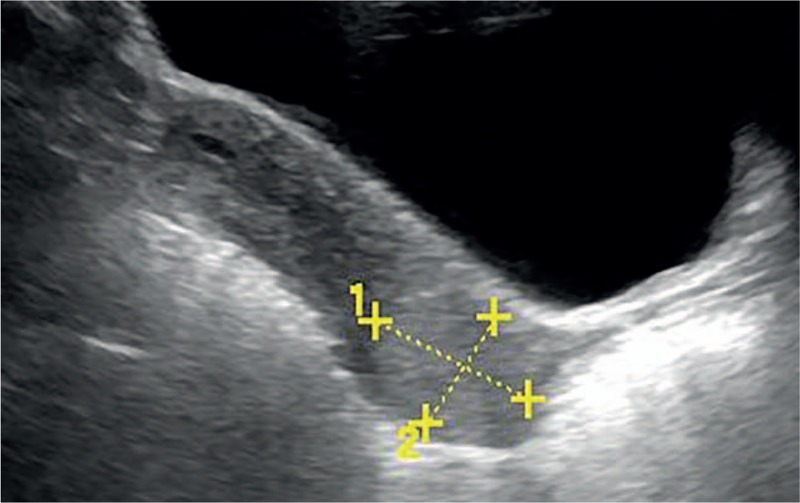
Transabdominal ultrasound imaging showing a well-circumscribed, strongly echogenic lesion in the cervix with endometrial cavity minimal fluid. The uterus was normal size. The endometrial thickness was 0.5 cm. No obvious abnormality was found in the double accessories. The lesion corresponded to an ETT on pathological examination.

**Figure 2:**
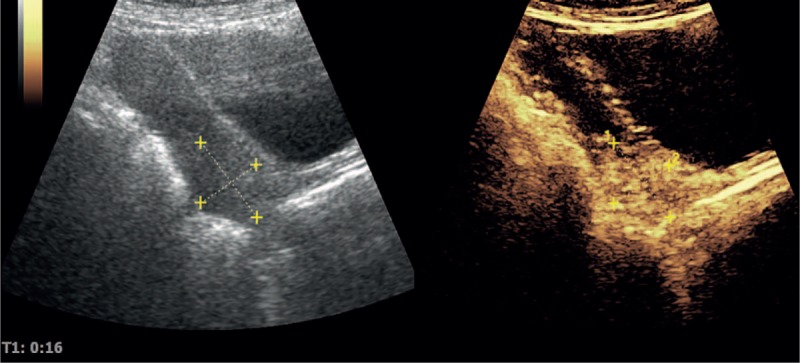
The enhanced pattern by contrast-enhanced ultrasound (CEUS) displayed strong peripheral enhancement accompanied with globular appearance and then centripetal filling completely.

A 60-year-old female presented to her primary physician with complaints of a 4-month history of irregular vaginal bleeding that had increased significantly over the previous 2 months. She had undergone dilatation and curettage for a missed miscarriage 35 years before diagnosis. Speculum examination showed a 5 cm friable exophytic mass on the cervix and touching bleeding. The biopsy was interpreted as poorly differentiated malignant neoplasm. Ultrasound (Voluson S8, General Electric Company, Gyeonggi, Korea) revealed a 3.0 cm × 2.7 cm well-circumscribed, strongly echogenic lesion in the cervix, with endometrial cavity minimal fluid (Fig. 1). The lesion was identified as a peripheral pattern of Doppler signals. The enhanced pattern by contrast-enhanced ultrasound (CEUS) displayed strong peripheral enhancement accompanied with globular appearance, then centripetal filling completely, and fading away rapidly (Fig. 2). Pelvic and abdomen magnetic resonance imaging (MRI) confirmed the presence of a well-circumscribed tumor in the same location, without evidence of local tissue infiltration or lymphadenopathy. MRI of her head and CT scan of chest did not reveal a metastatic disease. Although an unusual elevation of serum human chorionic gonadotropin (hCG) was noted (896.30 mIU/mL), there was no evidence to suggest pregnancy. Therefore, a presumptive diagnosis of tumor of IB2 cervical cancer was made. Surprisingly, after 2 courses neoadjuvant chemotherapy (NACT) of docetaxel, lobaplatin plus bleomycin, serum β-hCG revealed a rise up to 1582.0 mIU/mL. The patient was suspected to have ETT. A radical hysterectomy and bilateral salpingo-oophorectomy with bilateral pelvic lymph node dissection was performed.

On pathological examination, a yellow-brown mass of 2.5 cm in diameter was found in the cervix. Microscopic morphology consisted of crypt-liked tumor cells of diffuse infiltrative growth (type 1, 20%; adenocarcinoma) and polygonal atypical tumor cells arranged as nest with map-shaped necrosis (type 2, 80%; ETT). Immunohistochemically, tumor cells were strongly positive for CEA(+), GATA3(+), P63(+), P40(+), hCG(focal+), PCK(+), inhibin(focal+), CK8/18(+), E-ca(+), Ki67(+, 90%). Based on all these findings, the diagnosis was ETT accompanying middle-low differentiated mucinous adenocarcinoma. Postoperative recovery was uneventful. The serum β-hCG returned to normal range. The patient received 6 cycles systemic chemotherapy (paclitaxel liposome, lobaplatin plus/not plus bleomycin) and radiotherapy. At the time of reporting, the patient is currently disease-free for the past 1 year. But long-term follow-up with β-hCG levels is necessary for reducing recurrence rates.^[1]^

## 4 Discussion

Based on clinicopathology, GTN is classified into invasive mole (IM), choriocarcinoma (CC), placental-site trophoblastic tumor (PSTT), and ETT. In 1998, ETT was first characterized as a distinctive and rare subtype of GTN by Drs. Shih and Kurman,^[2]^ and subsequently received into the tumor classification of World Health Organization in 2003, which is commonly misdiagnosed. Most cases occur in reproductive-age women but also in postmenopausal women. The average latency period between the preceding gestation and initial diagnosis of ETT is 6.2 years (ranging from 1 to 18 years).^[2]^ The patients show the usually symptoms of vaginal bleeding and relatively low, but definitely elevated level of serum HCG without exceeding 2500 mIU/mL. Similar with PSTT, ETT is easy to chemoresistance. The recommended primary treatment is surgical intervention.^[3,4]^ Thus, in order to minimize the risk of mistreatment, an accurate diagnosis is indispensable.

Ultrasound is the initial imaging investigation when GTN is suspected in clinical routine.^[5,6]^ Since then, limited information available from the present studies regarding ultrasound imaging of ETT. There are no direct ultrasonographic signs to diagnose ETT, however, ultrasound can often identify uterine lesions. Most reported cases described the characteristic of ETT including a well-circumscribed tumor border surrounded by a hypoechogenic halo, growing in an expansive fashion and invading the cervix or myometrium deeply, which corresponds to our ultrasound findings.^[7–12]^ There is no uniform conclusion about the Color Doppler images of ETT lesions. Because of nonpenetrated by tumor cells, the intratumoral vessels of ETT are too small to be detected in Color Doppler image, or be just showed as low blood signals.^[13]^ Same with Qin and colleagues’ finding, we identified a Doppler signal spotted around the tumor instead of within the tumor. This was distinct from IM and CC. These sonographic findings were compatible with the expansive growth pattern and vascular morphology of typically ETT. Additionally, our case was composed primarily of trophoblastic component so that the ultrasonic characteristics of mucinous adenocarcinoma may not be quite as obvious.

CEUS has been demonstrated to significantly improve the detection of tumor perfusion and provide cleared and more accurate diagnosis information in comparison with B-mode US. Therefore, we examined CEUS for this patient. The enhanced pattern displayed strong peripheral enhancement accompanied with globular appearance, then centripetal filling completely, and fading away rapidly. The enhancement patterns are different between ETT and other GTN. Most of the latter ones showed diffuse and continue enhancement. It is valuable for diagnosis and differentiation for these diseases. The ultrasonographic features of the uterine lesions among ETT, PSTT, and IM/CC are listed in Table 1. Though the sample size is small and further studies with more samples are needed to validate these findings, our study suggests that ultrasound could discriminate ETT from other types of GTN.

**Table 1:**
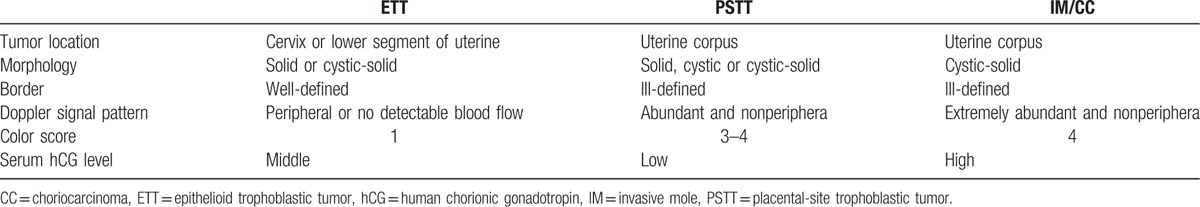
Ultrasonographic characteristics of the uterine lesion in ETT, PSTT, and IM/CC.

Approximately 50% of reported ETT arose from the uterine cervix or lower uterine segment. Histologically, it is a significant diagnostic challenge to separate ETT from an invasive squamous cell carcinoma (SCC) because of their cytological features and growth patterns similarity. However, the treatment of ETT is drastically different from those of SCC. The correct diagnosis is critical. Ultrasonography may be helpful for differentiating between these 2 types of tumor. Generally, sonographic image of SCC showed an ill-defined, low echogenic lesion in the cervix, which is different from ETT. An awareness of the typical clinical presentation with elevated serum hCG and/or well-circumscribed mass by ultrasound, the characteristic histological features and a panel of immunohistochemical markers are helpful in making a correct diagnosis.^[10]^

In conclusion, since the prognosis and treatment of ETT are drastically different from other GTN or SCC, a correct diagnosis is of paramount clinical relevance. It is meaningful for ultrasonographic diagnostic marker that ETT should be suspected when the well-circumscribed border in the uterine cervix or lower uterine segment with peripheral Doppler signal appears in patients with middle-level elevation of hCG and clinical symptoms. More cases need to be identified and published so that we may have a better understanding of this disease sonographic image.

## Acknowledgment

We thank the patient's families who agreed to publish the clinical data.
